# A novel, non-GMO surface display in *Limosilactobacillus fermentum* mediated by cell surface hydrolase without anchor motif

**DOI:** 10.1186/s12866-022-02608-9

**Published:** 2022-08-03

**Authors:** Robie Vasquez, Bernadette B. Bagon, Ji Hoon Song, Nam Soo Han, Dae-Kyung Kang

**Affiliations:** 1 Department of Animal Resources Science, Dankook University, 119 Dandae-ro, Cheonan, 31116 Republic of Korea; 2 Department of Food Science and Technology, Chungbuk National University, Cheongju, 361-763 Republic of Korea

**Keywords:** Heterologous display, Non-GMO, Cell surface display, Anchor domain, Lactic acid bacteria, Bacteria-like particle

## Abstract

Recent studies have demonstrated the potential of surface display technology in therapeutic development and enzyme immobilization. Utilization of lactic acid bacteria in non-GMO surface display applications is advantageous due to its GRAS status. This study aimed to develop a novel, non-GMO cell wall anchoring system for lactic acid bacteria using a cell-surface hydrolase (CshA) from *Lactiplantibacillus plantarum* SK156 for potential industrial and biomedical applications. Analysis of the CshA revealed that it does not contain any known classical anchor domains. Although CshA lacks a classical anchor domain, it successfully displayed the reporter protein superfolder GFP on the surface of several lactic acid bacteria in host dependent manner. CshA-sfGFP fusion protein was displayed greatest on *Limosilactobacillus fermentum* SK152. Pretreatment with trichloroacetic acid further enhanced the binding of CshA to *Lm. fermentum*. The binding conditions of CshA on pretreated *Lm. fermentum* (NaCl, pH, time, and temperature) were also optimized, resulting in a maximum binding of up to 10^6^ CshA molecules per pretreated *Lm. fermentum* cell. Finally, this study demonstrated that CshA-decorated pretreated *Lm. fermentum* cells tolerates gastrointestinal stress, such as low pH and presence of bile acid. To our knowledge, this study is the first to characterize and demonstrate the cell-surface display ability of CshA. The potential application of CshA in non-GMO antigen delivery system and enzyme immobilization remains to be tested.

**Supplementary Information:**

The online version contains supplementary material available at 10.1186/s12866-022-02608-9.

## Background

Surface display of foreign proteins has been around for four decades since the first expression system was designed using a “fusion phage” in the 1980s [1]. Since then, surface display applications have expanded to include microbial and fungal display hosts [2]. Currently, microbial display expression systems have been extensively studied for both gram-negative and gram-positive bacteria. Surface displays of foreign proteins have been exploited for the development of biocatalysts and biosensors [3–5]. Moreover, their use in biomedical applications such as live delivery systems for vaccines or antigens has also been explored [6–10]. To successfully display a foreign protein on the microbial cell surface, the protein of interest (POI) must first be fused (either at the amino or carboxyl terminus) with a peptide containing an anchor domain, which will facilitate surface display [2, 11–13]. Classical anchor domains include transmembrane anchors, lipoprotein anchors, LPXTG, LysM, WxL, and S-layer proteins, each with different binding ligands and mechanisms of attachment [11–13]. To date, there have been three commonly employed surface display strategies: (1) recombinant bacteria expressing and displaying the POI, (2) heterologous display of recombinant proteins on living cells, and (3) heterologous display of recombinant proteins on non-living cells or bacteria-like particles (BLPs, formerly gram-positive enhancer matrix or GEM) [11, 13, 14]. Although the recombinant approach may be advantageous because the display host can continuously express the POI [13], its GMO status poses serious concerns regarding its safety and market acceptability. In heterologous surface display strategies, the POI-anchor fusion is expressed in a different host (such as *E. coli*) and then displayed on the host’s cell surface; hence, the term heterologous display [14]. Heterologous approaches are categorized as non-GMO strategies, which are advantageous when regulatory and statutory limits are considered.

In gram-positive bacteria, non-GMO heterologous display of proteins is mostly performed using lactic acid bacteria (LAB). LAB are not only a great source of anchor proteins [12, 15] but are also commonly used as display hosts because of their generally regarded as safe (GRAS) status [13, 14, 16]. The implication of non-GMO surface displays on GRAS microorganisms is of great importance, especially in biomedical applications; hence, it is the most prevalent route for surface display studies [6]. Several studies on the use of LAB-displaying mucosal vaccines have shown promising results, which are proof-of-concept for the use of LAB-displaying proteins as an alternative vaccine delivery system [17–21]. Non-GMO surface displays with LAB have also been successfully applied to immobilize enzymes, as demonstrated in various studies [4, 22–24].

The increasing analytical power of bioinformatics tools and the accessibility of protein databases have helped researchers elucidate the components of the bacterial surfaceome [12]. This has provided researchers the opportunity to explore the surfaceome for novel anchor domains. Kleerebezem et al. [16] reported that *Lp. plantarum* contains the greatest number of exoproteomes among LAB, most of which are anchored to the cell surface, such as cell surface hydrolases. The objective of this study was to develop a novel non-GMO cell wall anchoring system for LAB. A new cell-surface anchor, herein designated as CshA, was discovered in the lp_3265 gene of *Lactiplantibacillus plantarum* SK156, which encodes a putative cell-surface hydrolase in the genome. The ability of CshA to bind to the LAB surface was examined by appending a reporter protein, superfolder green fluorescent protein (sfGFP) [25]. Optimization of CshA binding was performed, and its stability in a simulated gastrointestinal tract (GIT) environment was challenged. This study is the first to characterize CshA and demonstrate its surface-anchoring ability on LAB.

## Results

### Characterization of the putative cell-surface hydrolase CshA

**Fig. 1:**
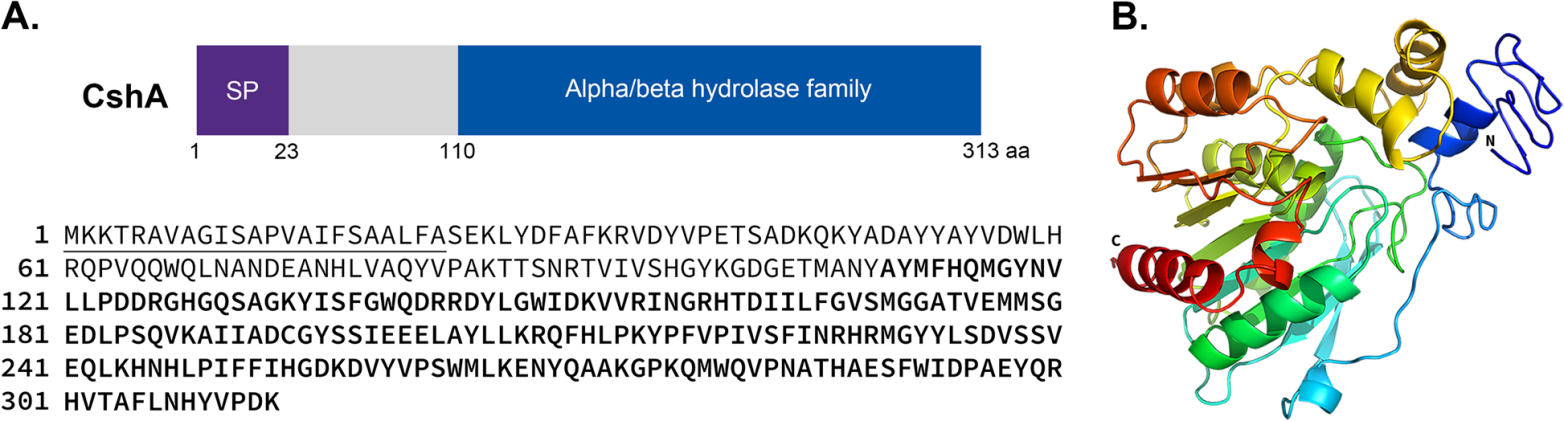
Structure and sequence of the putative cell-surface hydrolase, CshA. Schematic diagram of the CshA, and its amino acid sequence (**A**). The sequence for the signal peptide (SP) is underlined, while the sequence for the active site containing the alpha/beta hydrolase is in bold. Three-dimensional structure of CshA rendered by I-TASSER showing the alternating α-helices and β-strands (**B**)

A putative cell-surface hydrolase (lp_3265) was identified in the genome of *L. plantarum* SK156 (939 bp). It was selected from a pool of anchor candidates identified from *Lp. plantarum* SK156 (data not shown). The expressed protein had 313 amino acid (aa) residues and a mass of 36 kDa. It has a predicted signal peptide sequence at the N-terminal (1–23 aa) (Fig. 1a; Supplementary Figure S1), whereas the C-terminal was predicted by Pfam and InterPro to belong to the alpha/beta hydrolase superfamily (110–313 aa) (Fig. 1A). However, Pfam and InterPro searches were unable to identify any known anchor motifs in the protein sequence of the putative protein. Structural analysis of the putative protein using I-TASSER revealed that it contains alternating α-helices and β-strands, whereas functional prediction revealed that the putative protein has a hydrolytic function (Fig. 1B). A BLASTp search showed that the putative hydrolase can be found almost exclusively in the genus *Lactiplantibacillus* (99%–100% similarities), and some *Lactobacillus* and *Loigolactobacillus* (Supplementary Figure S2).

### Surface display of CshA-sfGFP on LAB

**Fig. 2:**
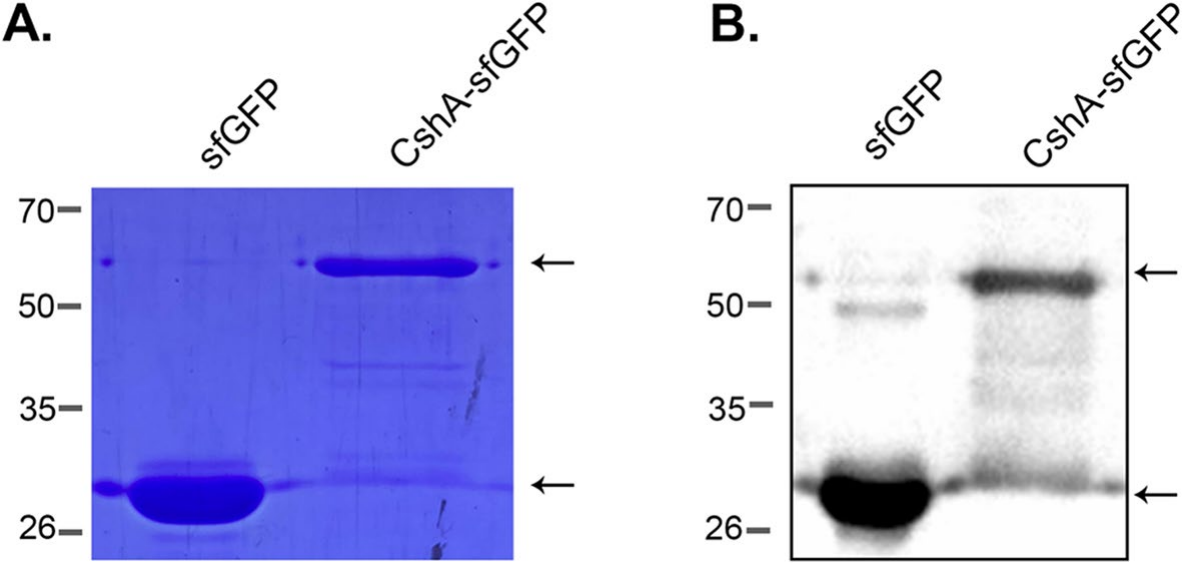
Expression of the sfGFP and CshA-sfGFP proteins. Overexpression of the sfGFP (28 kDa) and CshA-sfGFP (63 kDa) was confirmed through sodium dodecyl sulfate–polyacrylamide gel electrophoresis (SDS-PAGE) (**A**), and Western blot (**B**). (Gels and blots were cropped for clarity. The full-length images are included in the Additional file, Figure S3)

**Fig. 3:**
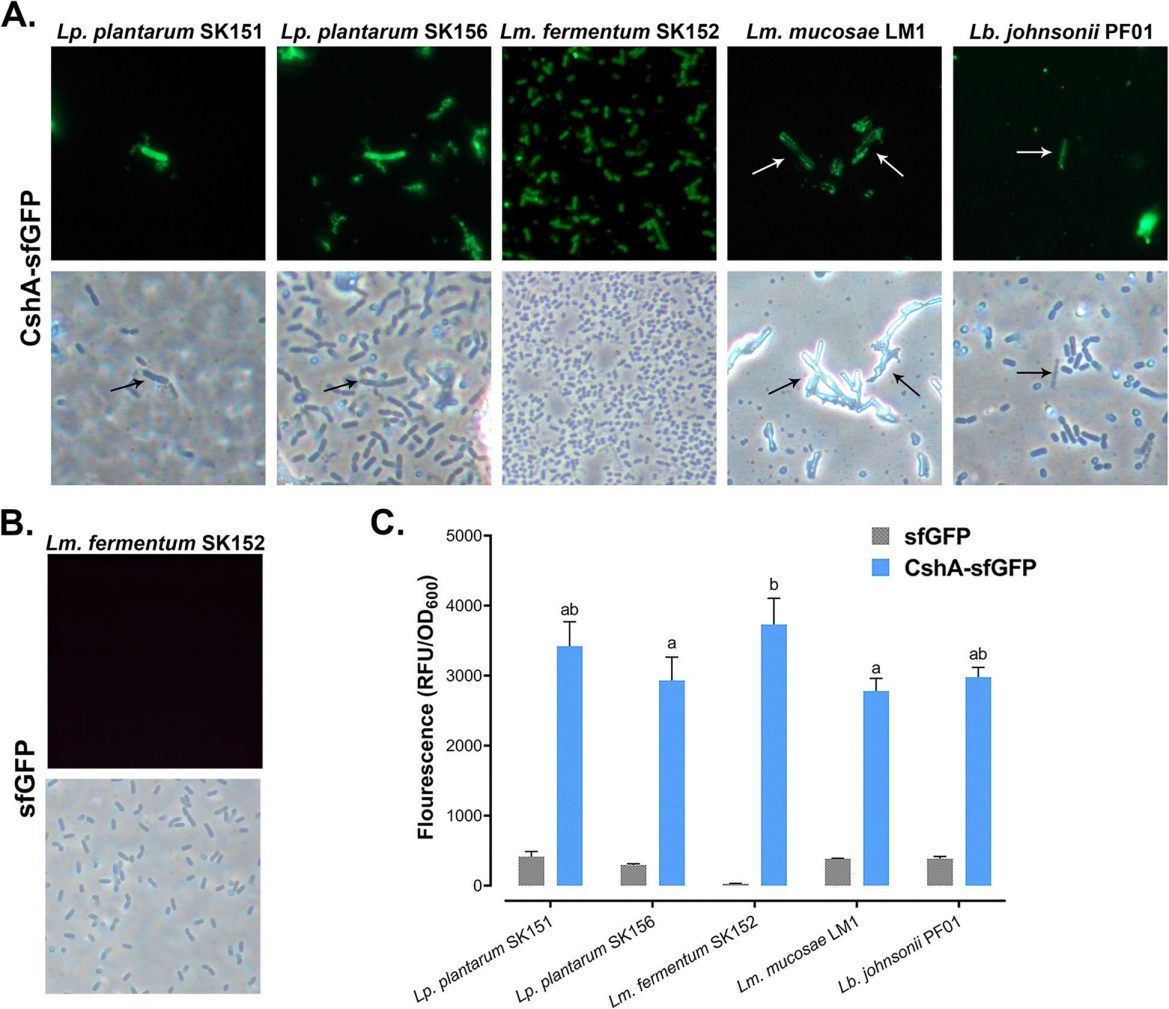
Localization of the CshA-mediated surface display of sfGFP on LAB. Surface display of CshA on *Lactiplantibacillus plantarum* SK151, *Lp. plantarum* SK156, *Limosilactobacillus fermentum* SK152, *Lm. mucosae* LM1, and *Lactobacillus johnsonii* PF01 (**A**). Cell-associated fluorescence was observed using fluorescence microscopy (top row). sfGFP alone cannot bind to the cell surface of *Lm. fermentum* (**B**). Surface binding of CshA to LAB is host-dependent, with preference to *Lm. fermentum* (**C**). Significant differences were determined using ANOVA with Tukey’s test for pairwise comparison of means and denoted by difference in letters

To test the ability of CshA to display the reporter protein, CshA-sfGFP (64 kDa) and sfGFP (28 kDa) were overexpressed and purified, as shown in Fig. 2A. Western blot analysis confirmed the presence of both proteins (Fig. 2B) and was subsequently used for the binding experiment. CshA-sfGFP was successfully displayed on all LAB strains, albeit with different capacities (Fig. 3A). In addition, CshA-sfGFP demonstrated greater display of *Lm. fermentum* SK152 compared with other LAB strains (Fig. 3C). Surprisingly, CshA showed relatively low binding to *Lp. plantarum* SK156, the protein source and even on a similar species, *Lp. plantarum* SK151 than *Lm. fermentum* SK152 (*P* > 0.05 and *P* < 0.05, respectively). In addition, the binding of CshA to *Lm. mucosae* LM1 and *Lb. johnsonii* PF01 was limited and non-uniform. This suggests that the binding of CshA is host dependent. As a negative control, sfGFP did not bind to *the Lm. fermentum* (Fig. 3B), indicating that CshA is necessary to display the sfGFP protein on the surface of LAB cells. Based on these results, *the Lm. fermentum* was chosen as the display host for the subsequent binding experiments.

### Pretreatment of *Lm. fermentum* cells increased binding of CshA

**Fig. 4:**
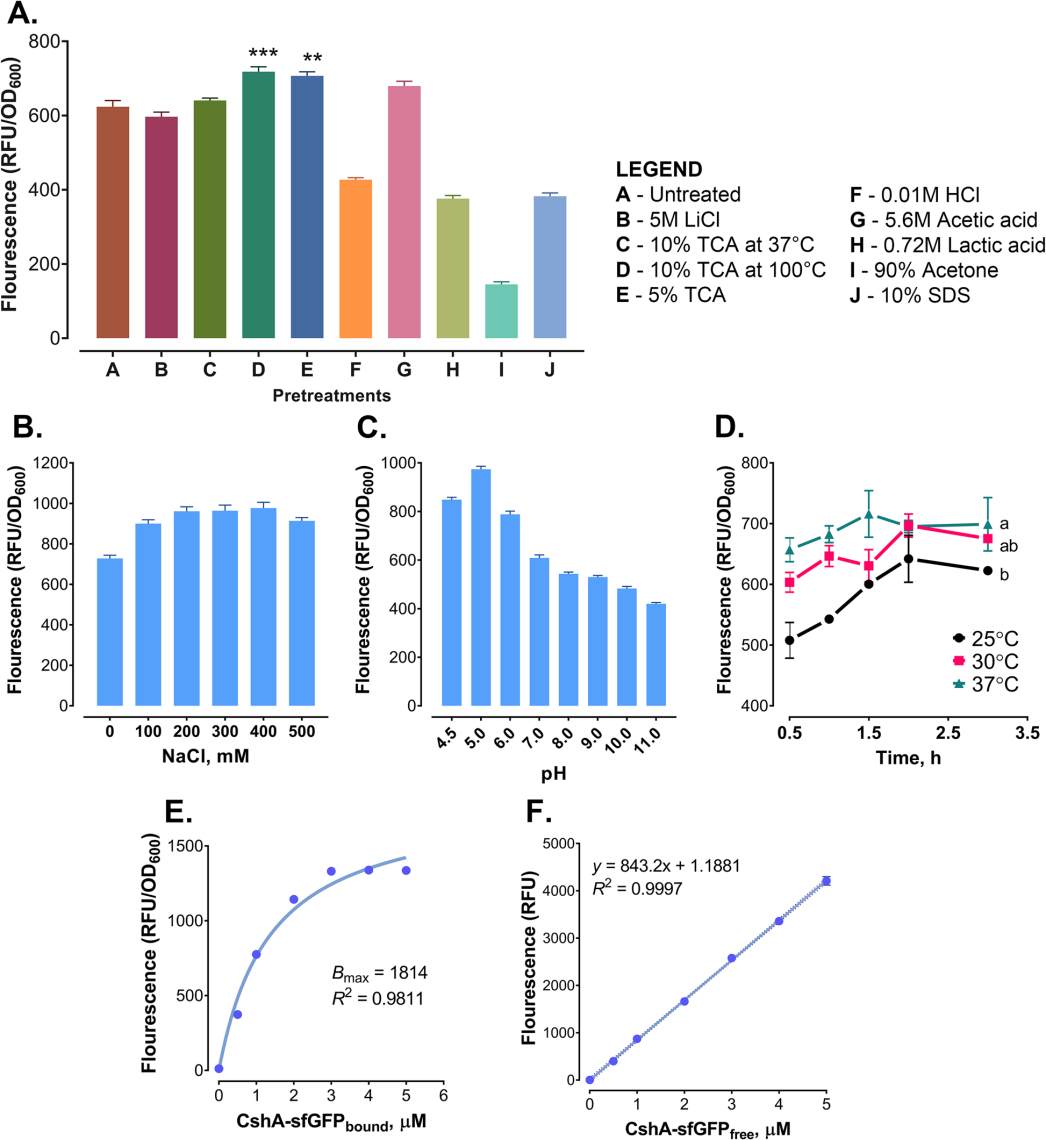
Optimization of the CshA binding on *Lm. fermentum* cells. Pretreatment of the *Lm. fermentum* cells affected the surface binding of CshA (**A**). NaCl concentration (**B**) and pH (**C**), as well as the binding time and temperature (**D**) also influenced the surface binding of CshA. The binding capacity of pretreated *Lm. fermentum* cells was determined by fitting the fluorescence at different protein concentration into a nonlinear curve (**E**), then calculated using a standard curve (**F**). All experiments were done in triplicates and reported as mean ± SD. Significant differences were determined using ANOVA with Tukey’s test for pairwise comparison of means. Differences are denoted by ** *P* < 0.01 and *** *P* < 0.001, or by different letters

To examine the effect of pretreatment on the binding ability of CshA, *Lm. fermentum* was exposed to different chemical agents that removed cell wall components (Fig. 4A). CshA showed a higher binding preference (15% increase) in cells pretreated by boiling with 10% or 5% trichloroacetic acid (TCA) than in untreated cells (*P* < 0.001 and *P* < 0.01, respectively). Pretreatment with 0.01 M hydrochloric acid (HCl), 0.72 M lactic acid, 90% acetone, and 10% sodium dodecyl sulfate (SDS) resulted in a decrease in fluorescence intensity compared with the untreated cells. No significant changes were observed in the binding of CshA to cells treated with either 5 M lithium chloride (LiCl), 10% TCA (37 °C), or 5.6 M acetic acid. Considering these results, it is likely that CshA targets the peptidoglycan layer of the cell wall. Therefore, 5% TCA was used in subsequent binding experiments.

### Optimization of CshA binding to pretreated *Lm. fermentum* cells

To further augment cell-surface binding of CshA to pretreated *Lm. fermentum*, binding conditions for CshA, such as NaCl concentration, pH, time, and temperature, were optimized. Display of the CshA-sfGFP fusion protein in pretreated *Lm. fermentum* cells were performed under different NaCl concentrations and pH levels (Fig. 4B, C). Fluorescence intensity increased as the NaCl concentration increased and then plateaued at 200–400 mM before decreasing at 500 mM, suggesting that optimal binding can be achieved at approximately 300 mM NaCl. Meanwhile, the fluorescence intensity of the CshA-sfGFP-decorated cells peaked at pH 5 and then started to decrease at pH 6, indicating that the optimal binding is at a slightly acidic pH of 5. At 30 °C and 37 °C, binding of CshA to TCA-pretreated *Lm. fermentum* cells were achieved within 2 h (Fig. 4D). The binding of CshA was significantly reduced at 25 °C even after incubation for 3 h (*P* < 0.05).

To determine the maximum CshA-binding capacity of the pretreated *Lm. fermentum*, binding experiments were performed using different concentrations of CshA proteins. It was observed that the fluorescence intensity increased with concentration before plateauing at approximately 3 µM. The data were fitted to a nonlinear curve using a single-site binding model, resulting in *a B*_max_ of 1814 RFU (Fig. 4E). Using a standard curve (Fig. 4F), the corresponding CshA protein concentration was determined to be 2.15 µM. This indicates that *Lm. fermentum* cell at optical density of ~ 1.8 at 600 nm (OD_600_) (approximately 10^8^) can display 27 µg of CshA-sfGFP fusion protein, or around 2.2 × 10^6^ fusion protein molecules per cell.

### Heterologous display via CshA can be retained in GIT conditions

**Fig. 5:**
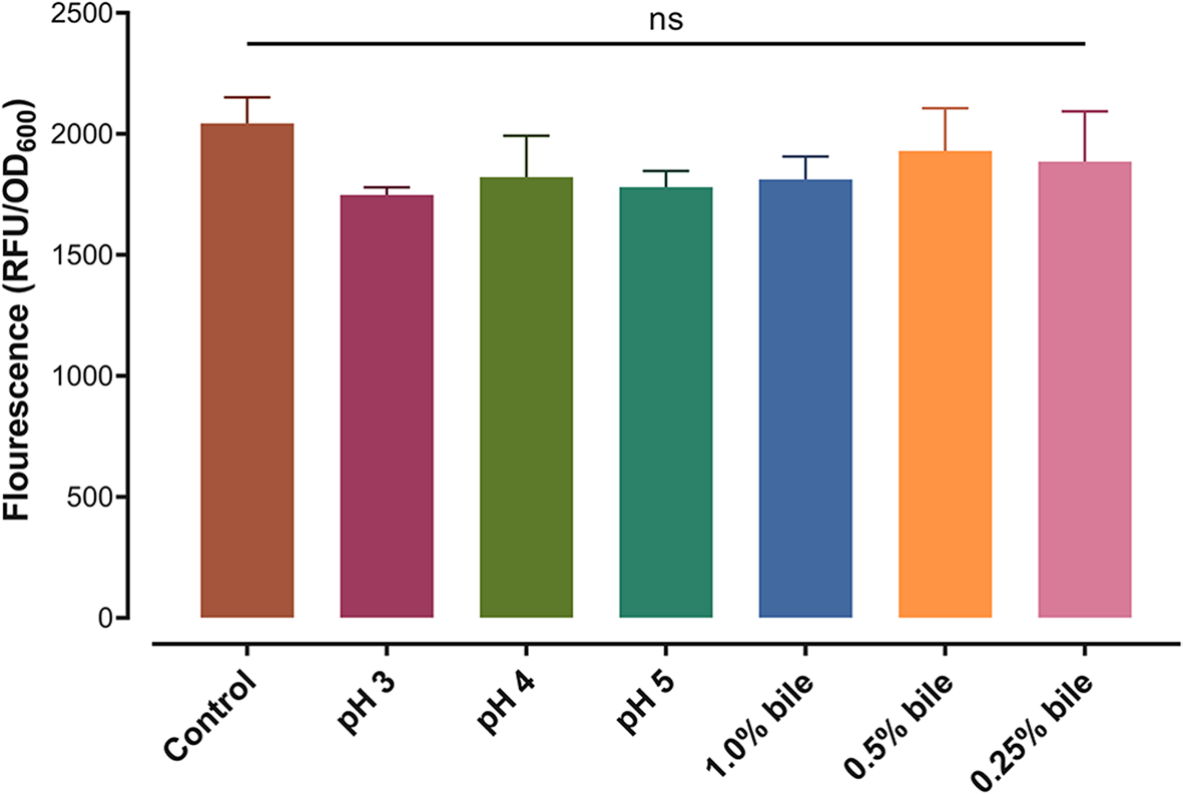
CshA-mediated surface display on pretreated *Lm. fermentum* showed stability under simulated gastrointestinal tract conditions. There was no significant (ns) loss of fluorescence at different pH (3–5) and bile concentrations (0.25%–1%) compared with the control setup. All experiments were done in triplicates and reported as mean ± SD. Significant differences were determined using ANOVA with Tukey’s test for pairwise comparison of means

The display stability of the CshA-sfGFP fusion protein under conditions mimicking the gastrointestinal environment was investigated. As shown in Fig. 5, the display of the fusion protein was retained with no significant loss of cell-associated fluorescence at pH 3–5 and bile concentrations of 0.25%–1% compared with the control setup (*P* > 0.05). This suggested that the binding of CshA to TCA-pretreated *Lm. fermentum* can tolerate harsh gastrointestinal conditions and is potentially applicable as a non-GMO oral delivery system.

## Discussion

The utilization of LAB in heterologous surface displays is of great interest to researchers because of its GRAS status and potential in non-GMO applications [13]. Moreover, the potential of LAB in surface displays for biocatalytic applications and oral vaccine delivery systems has been demonstrated in several studies [4, 10, 20, 21]. LysM, LPXTG, and S-layer protein domains are some of the most common anchors used in heterologous displays in LAB [2, 10, 11]. In this study, we investigated CshA, a putative cell-surface hydrolase identified in *Lp. plantarum* SK156 as a novel anchoring domain. CshA belongs to the alpha/beta hydrolase superfamily and contains an N-terminal SP. These extracellular alpha/beta hydrolases are ubiquitous in LAB, but their specific functions are poorly understood [15]. Our BLASTp analysis revealed that CshA is present only in *Lactiplantibacillus* and some *Lactobacillus* and *Loigolactobacillus* species, suggesting that its function is specific to these genera of LAB. As with most hydrolases present in the bacterial surfaceome, it was initially thought that CshA may contain a cell wall-binding domain, unlike the LysM domain-containing AcmA [26, 27]. However, Pfam and InterPro sequences revealed that CshA did not contain any known anchor motifs. Nevertheless, CshA displayed the reporter protein sfGFP in different LAB species. This strongly proves that CshA can successfully display proteins on the surface of LAB, despite the lack of anchoring motifs. In recent studies, ‘anchorless’ extracellular LAB proteins have been described [12, 28, 29]. Glenting et al. [30] showed that the glycolytic enzymes GADPH and enolase from *Lp. plantarum* are bound to the cell surface without anchor motifs. Similarly, Mu et al. [24] demonstrated the surface display ability of EnoM, an enolase from *Streptococcus thermophilus,* despite it being devoid of conserved anchor domains. Proteins with additional non-enzymatic functions are referred to as moonlighting proteins, which have been reported to play a role in host mucosal adhesion and colonization [16, 29–31]. Moonlighting proteins do not rely on the anchor domain for binding; instead, they bind to cell-surface components (teichoic acid or peptidoglycan) through ionic interactions or at low pH [12, 32, 33]. Similarly, binding of CshA to pretreated *Lm. fermentum* was affected by NaCl concentration and was maximum at low pH, suggesting that these factors play crucial roles in the molecular interaction between CshA and its binding ligand. Whether CshA is a moonlighting protein, or it contains a binding domain yet to be identified and requires further investigation.

In the current study, the binding of CshA to LAB demonstrated host preference, specifically for *Lm. fermentum* cells. This phenomenon of host-dependent binding has been observed in other studies [22, 34], owing to the differences in cell wall components of these LAB, such as capsular polysaccharides on *Lp. plantarum* and S-layer proteins on *Lb. johnsonii*, which protects the cell wall from heterologous binding to CshA. The surface of *Lm. fermentum* has been reported to contain fewer proteins than *Lp. plantarum* [16], which may explain the higher binding preference of CshA. This suggests that the surface of *Lm. fermentum* offers less resistance to the binding of CshA, which makes it a good host for surface display.

Anchor domains are known to either covalently or non-covalently bind to cell wall components such as peptidoglycan or cell-surface proteins [11, 13, 35]. To investigate the binding target of CshA, we first exposed *Lm. fermentum* cells using different chemical agents to remove the components of the cell wall. The binding of CshA increased by 15% after TCA treatment. Boiling cells in TCA for 10 min removes teichoic acid and surface proteins and exposes the peptidoglycan layer [14, 35]. On the other hand, other pretreatments had either no effect or reduced the binding of CshA. LiCl and SDS remove surface layer proteins while acetone removes cell wall associated proteins [14, 22, 34]. Meanwhile, hydrochloric, acetic, and lactic acid have also been used previously to alter the cell wall components [23, 35]. This potentially suggests that the peptidoglycan layer may be the target substrate of CshA, although specific cell wall proteins may also be interacting with CshA. Cell pretreatment, especially with TCA, is a common strategy to enhance the binding of anchors to the cell surface of gram-positive bacteria, thereby creating BLPs [23, 35, 36]. BLPs have been widely used as display hosts for proteins, particularly for oral vaccine delivery [14]. The potential applications of CshA-decorated *Lm. fermentum* BLPs must be explored in the future.

Owing to its GRAS status and potential in non-GMO applications, LAB have been exploited as an alternative delivery vector for prophylactic and therapeutic molecules via a surface display approach [6, 14]. However, the hostile environment of the GIT presents a challenge, both for the stability of the heterologous display and for the activity of the POI as it traverses the GIT. Thus, determining the stability of the surface display of CshA under simulated GIT stress is challenging. It was observed that the surface display of the CshA-sfGFP fusion protein on pretreated *Lm. fermentum* cells was stable at gastric pH (3–5) and high bile concentration (0.25%–1%). This indicated the potential of CshA as a non-GMO delivery vector system. However, careful selection of POI to be displayed via CshA must be performed, as different proteins with or without anchor-POI fusions behave differently under various conditions (e.g., acid and bile-labile proteins) [21, 37].

## Conclusion

In summary, we characterized CshA, a putative cell-surface hydrolase, as a novel anchoring system for *Lp. plantarum* SK156. Despite the lack of known classical anchor motifs, CshA demonstrated excellent binding to the LAB surface, particularly to *Lm. fermentum* SK152. TCA pretreatment of *Lm. fermentum* cells, and optimization of the binding conditions significantly enhanced the binding ability of CshA (up to 10^6^ molecules of CshA per pretreated *Lm. fermentum* cells) compared with untreated cells. Finally, we demonstrated that the CshA-mediated heterologous surface display was stable in a GIT-simulated environment. Therefore, we conclude that CshA is a viable candidate as a non-GMO anchoring system. The applicability of CshA in enzyme immobilization and oral drug delivery or vaccine development is yet to be demonstrated in future investigations.

## Methods

### Bioinformatic analyses on CshA

The whole genome of *Lp. plantarum* SK156 (Genbank Accession No. CP059473) was analyzed by Hwang et al. [38]. Identification of classical anchor domains was performed using the Pfam [39] and InterPro [40] databases. Subcellular localization and the presence of signal peptides (SP) were predicted using SignalP v 6.0 [41]. Protein structural and functional predictions were performed using the I-TASSER online server [42–44]. BLASTp was used to compare the amino acid sequences against those of other bacteria (https://blast.ncbi.nlm.nih.gov/BLAST).

### Bacterial strains and culture conditions

The bacterial strains used in this study are listed in Table 1. *E. coli* DH5α and *E. coli* BL21 (DE3) were used as cloning and expression hosts, respectively. *E. coli* strains were grown in Luria–Bertani (LB) broth (BD Difco, USA) supplemented with ampicillin (100 µg/mL) at 37 °C with aeration. LAB strains were cultured in Man Rogosa Sharpe (MRS) broth (BD Difco, USA) at 37 °C without aeration.

**Table 1:** Bacterial strains, plasmids, and primers used in this study

	**Features or sequences**	**Source**
**Strains**		
*Escherichia coli* DH5α	Cloning host; F- endA1 glnV44 thi-1 recA1 relA1 gyrA96 deoR nupG Φ80dlacZΔM15 Δ(lacZYA-argF) U169, hsdR17(rK- mK +), λ–	Biofact
*E. coli* BL21 (DE3)	Expression host; F- *ompT hsdS*B(rB-mB-) *gal dcm* (DE3)	Real BioTech
*Lactiplantibacillus plantarum* SK151	Display host, wild type	Our laboratory
*Lp. plantarum* SK156	Display host, wild type	Our laboratory
*Limosilactobacillus fermentum* SK152	Display host, wild type	Our laboratory
*Lm. mucosae* LM1	Display host, wild type	Our laboratory
*Lactobacillus johnsonii* PF01	Display host, wild type	Our laboratory
**Plasmids**		
pET21b ( +)	Expression vector; N-terminal 6His-tag, Amp^r^	
pSfGFP	pET21b ( +) carrying 6 His-tagged sfGFP gene, Amp^r^	This study
pCSHA-sfGFP	pET21b ( +) carrying 6 His-tagged cshA-sfGFP fusion gene, Amp^r^	This study
pCB4270B-sfGFP	Plasmid containing sfGFP gene	[45]
**Primers**		
C1	5′-CCCCATATGAAAAAAACACGCGCC-3′	This study
C2	5′-ACCCTTTGACATGCGTTT**ATCAGGAACATAGTG**-3′	This study
CS1	5′-**GTTCCTGATAAACGC**ATGTCAAAGGGTGAAGAA-3′	This study
CS2	5′-GGGCTCGAGCTTGTATAATTCATCCATACC-3′	This study
S1	5′-GGGGCTAGCATGTCAAAGGGTGAAGAA-3′	This study
S2	5′-GGGCTCGAGCTTGTATAATTCATCCATACCATG-3′	This study

Nucleotide sequences in bold are overlapping sequences for fusion PCR

Enzyme restriction sites are underlined accordingly: *Nh*eI and *Xh*oI

### Molecular cloning

The plasmids and PCR primers used in this study are listed in Table 1. All PCRs were performed using *Taq* polymerase (TaKaRa, Tokyo, Japan). The *cshA* and *sfGFP* genes were amplified from the chromosomal DNA of *Lp. plantarum* SK156 and pCB4270B-sfGFP plasmids [45], respectively, using primers C1 and C2 (*cshA*), CS1 and CS2 (*sfGFP* with overlap), and S1 and S2 (*sfGFP* only). Amplicons were excised and cleaned from the agar gel using a NucleoSpin® Gel and PCR Clean-up Kit (Machery-Nagel, Düren, Germany). To generate *cshA-sfGFP*, purified *cshA* and *sfGFP* amplicons were used as templates for overlap PCR using the primers C1 and CS2. The enzyme restriction and ligation (T4 ligase) reactions were performed according to the manufacturer’s instructions (TaKaRa, Tokyo, Japan). The PCR products, *sfGFP* and *cshA-sfGFP*, were digested with *Nhe*I and *Xho*I, and then ligated into the *Nhe*I*/Xho*I sites of pET21b ( +) to construct pSfGFP and pCSHA-sfGFP, respectively. To check for sequence correctness, *E. coli* DH5α was transformed with either pSfGFP or pCSHA-sfGFP, according to the manufacturer’s protocol (Biofact, Daejeon, Republic of Korea). For protein overexpression, *E. coli* BL21 (DE3) was transformed with either pSfGFP or pCSHA-sfGFP according to the manufacturer’s protocol (Real BioTech, Taipei, Taiwan).

### Protein overexpression and purification

*E. coli* BL21 (DE3) cells harboring either pSfGFP or pCSHA-sfGFP were grown overnight in LB broth supplemented with ampicillin (100 µg/mL) at 37 °C with aeration. Overnight cultures were then diluted 1:100 in LB broth with ampicillin and allowed to grow to an OD_600_ of 0.6. Protein overexpression was induced by adding 0.1 mM isopropyl-β-D-thiogalactopyranoside (IPTG) to the culture. After incubation at 25 °C for 6 h, cells were harvested by centrifugation at 10,000 × *g* for 10 min and then washed twice with phosphate buffer saline (PBS; pH 7). Cell pellets were resuspended in lysis buffer (50 mM Tris, 300 mM NaCl, and 1 mM phenylmethylsulfonyl fluoride [PMSF], pH 8) and disrupted using a sonicator for 6–7 cycles (10 s sonication, 15 s pause) on ice. After sonication, the clear lysate (for sfGFP protein) or pellet (for CshA-sfGFP protein) was collected by centrifugation at 13,000 × *g* for 20 min. The clear lysate was filtered using a 0.22-µm filter to remove cell debris, and the cell pellet was first solubilized with 8 M urea and then passed through a 0.22-µm filter. His-tag protein purification was performed as described by Spriestersbach et al. [46] under native conditions for the sfGFP protein or denaturing conditions for the CshA-sfGFP protein. Purified proteins were dialyzed in a protein storage buffer (50 mM Tris, 150 mM NaCl, 1 mM dithiothreitol, 30% glycerol, pH 8). The purified proteins were stored at − 20 °C until further use.

### SDS-PAGE and western blotting

Bradford protein assay was performed to determine protein concentration (Bio-Rad, Germany). Protein expression was confirmed by sodium dodecyl sulfate–polyacrylamide gel electrophoresis (SDS-PAGE). Gels were stained with Coomassie blue or transferred onto a 0.45-µm nitrocellulose membrane (Bio-Rad, Germany) at 400 mA for 90 min for western blot analysis. After transfer, the membrane was washed thrice with TBST (1 × Tris-buffered saline 0.1% Tween 20) and blocked with 5% bovine serum albumin (BSA) in TBST for 1 h at room temperature. Anti-His antibody (1:10,000 dilution in TBST with 2% BSA) was added as the primary antibody and incubated overnight at 4 °C with slight agitation. After exposure to the primary antibody, the membrane was washed thrice before incubation with HRP-conjugated anti-His antibody (Thermo Scientific, USA) for 1 h at room temperature. Detection was carried out using the SuperSignal® West Pico Chemiluminescent Substrate kit (Thermo Scientific, USA), following the manufacturer’s instructions, and then visualized with ChemiDoc™ XRS + and Image Lab™ software (Bio-Rad, Germany).

### Surface display of CshA-sfGFP on LAB

Overnight cultures of LAB species were prepared for the binding experiments. One milliliter of each LAB culture was collected, centrifuged at 8000 × *g* for 10 min, and washed twice with PBS (pH 7). Harvested cells were incubated with either purified CshA-sfGFP or sfGFP proteins in binding buffer (1 × PBS, pH 7) at 37 °C for 2 h. Next, cells were collected by centrifugation at 10,000 × *g* for 5 min and washed twice with the binding buffer. The fluorescence intensity was determined using a spectrophotometer (SpectraMax, Molecular Diagnostics, USA) with excitation at 485 nm and emission at 511 nm. Cell background fluorescence was determined as relative fluorescence units (RFU). The fluorescence intensity was normalized by dividing the RFU values by OD_600_. The cell-surface display was visualized using a Nikon Eclipse 80i with a GFP filter (Nikon, New York, USA).

### Surface display of CshA-sfGFP on pretreated *Lm. fermentum* cells

Chemical pretreatment of the cell surface of *L. fermentum* was performed according to previously described methods [22, 23, 47]. Briefly, 1 mL of overnight *Lm. fermentum* cultures were harvested by centrifugation at 8000 × *g* for 10 min and washed twice with PBS (pH 7). Harvested cells were treated with the following chemicals and conditions: 5 M LiCl and 10% TCA at 37 °C for 1 h; 10% TCA, 5% TCA, 0.01 M HCl, 5.6 M acetic acid, 0.72 M lactic acid and 10% SDS at 100 °C for 10 min; and 90% acetone at room temperature for 10 min. Cells were collected and washed twice with PBS to remove residual chemicals prior to binding experiments.

### Factors affecting the display of CshA-sfGFP on *Lm. fermentum*

*Lm. fermentum* was grown overnight in MRS broth until it reached an OD_600_ of ~ 1.8. Cell cultures were prepared and pretreated with 5% TCA as described above. To investigate the effect of NaCl concentration and pH on the display of CshA-sfGFP, a binding experiment was performed using binding buffer with either varying concentrations of NaCl (0, 100, 200, 300, 400, and 500 mM) or varying pH levels (4.5–11). To determine the optimal binding temperature and time, binding experiments were performed at different temperatures (25, 30, and 37 °C) at different time points (0.5, 1, 1.5, 2, and 3 h).

### Binding capacity of CshA on *Lm. fermentum*

To determine the binding capacity of CshA to *Lm. fermentum*, the methods from Tay et al. [22] were adapted for this study. Briefly, the binding experiment was performed with different concentrations of CshA-sfGFP protein (0, 0.5, 1, 2, 3, 4, and 5 µM). The relative fluorescence values for each point were determined and fitted to a nonlinear curve, and the *B*_max_ and *R*^2^ values were calculated. A standard curve using the free CshA-sfGFP protein was created to determine the protein concentration at a specific *B*_max_ value. Uniformity of the distribution of bound proteins in the cells was assumed.

### Surface display retention of CshA-sfGFP on *Lm. fermentum* under various conditions

To test the display retention of CshA-sfGFP on *Lm. fermentum*, the method described by Gordillo et al. [37] was performed with modifications. The binding experiments were performed as described above. Pretreated *Lm. fermentum* cells displaying CshA-sfGFP were collected and subsequently incubated in PBS at varying pH levels (3–5) or bile salt concentrations (0.25, 0.50, and 1%) at 37 °C for 2 h to simulate the conditions of the GIT. As a control, the CshA-decorated *Lm. fermentum* were incubated in PBS at pH 7 without bile salts. After incubation, the cells were washed twice and collected to determine the fluorescence intensity.

### Statistical analyses

All statistical analyses in this study were performed using GraphPad Prism version 8.4.2 for Windows (GraphPad Software, San Diego, California, USA). One-way ANOVA with Tukey’s test was performed to determine significant differences in the binding studies. Differences were considered statistically significant at *P* < 0.05. Nonlinear regression was performed to calculate *B*_max_ using the one-site binding model in GraphPad Prism. All experimental assays were performed in triplicate. All values are reported as mean ± standard deviation (SD).

## Supplementary Information

**Additional file 1: Figure S1. **Signal peptide (SP) prediction using SignalP v6.0. **Figure S2.** BLASTp analysis shows that CshA is present in *Lactiplantibacillus, Lactobacillus *and *Loigolactobacillus* genera. **Figure S3.** Full-length images for the SDS-PAGE and western blot, including replicates. M, marker; 1, sfGFP; 2,CshA-sfGFP. No enhancements were done to the images.

## Data Availability

The whole genome sequence of *Lp. plantarum* SK156 used in this study can be accessed from NCBI Genbank, https://www.ncbi.nlm.nih.gov/nuccore/CP059473. Other datasets used and/or analyzed during the current study are available from the corresponding author upon reasonable request.

## Abbreviations

BLP: Bacteria-like particle
GIT: Gastrointestinal tract
GEM: Gram-positive enhancer matrix
GMO: Genetically modified organism
GRAS: Generally recognized as safe
LAB: Lactic acid bacteria
RFU: Relative fluorescence unit
SDS-PAGE: Sodium dodecyl sulfate polyacrylamide gel electrophoresis
sfGFP: Superfolder green fluorescent protein
TCA: Trichloroacetic acid
