# Supplementary material for: A novel, non-GMO surface display in Limosilactobacillus fermentum mediated by cell surface hydrolase without anchor motif
Source: BMC Microbiol. 2022 Aug 3;22:190. doi: 10.1186/s12866-022-02608-9 (PMC9347134; doi:10.1186/s12866-022-02608-9)
Supplement: Supplementary file 1 — Additional file 1: Figure S1. Signal peptide (SP) prediction using SignalP v6.0. Figure S2. BLASTp analysis shows that CshA is present in Lactiplantibacillus, Lactobacillus and Loigolactobacillus genera. Figure S3. Full-length images for the SDS-PAGE and western blot, including replicates. M, marker; 1, sfGFP; 2,CshA-sfGFP. No enhancements were done to the images. [file 12866_2022_2608_MOESM1_ESM.docx]

**Additional file**

**A novel, non-GMO surface display in *Limosilactobacillus fermentum* mediated by cell surface hydrolase without anchor motif**

Robie Vasquez^1^, Bernadette B. Bagon^1,#^, Ji Hoon Song^1^, Nam Soo Han^2^, and Dae-Kyung Kang^1,^*


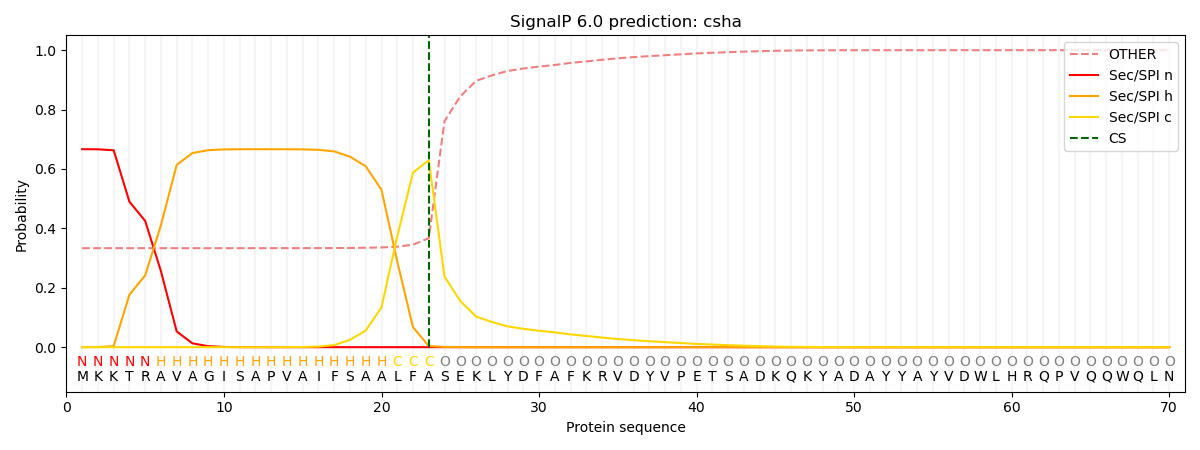


**Figure S1.** Signal peptide (SP) prediction using SignalP v6.0.

**
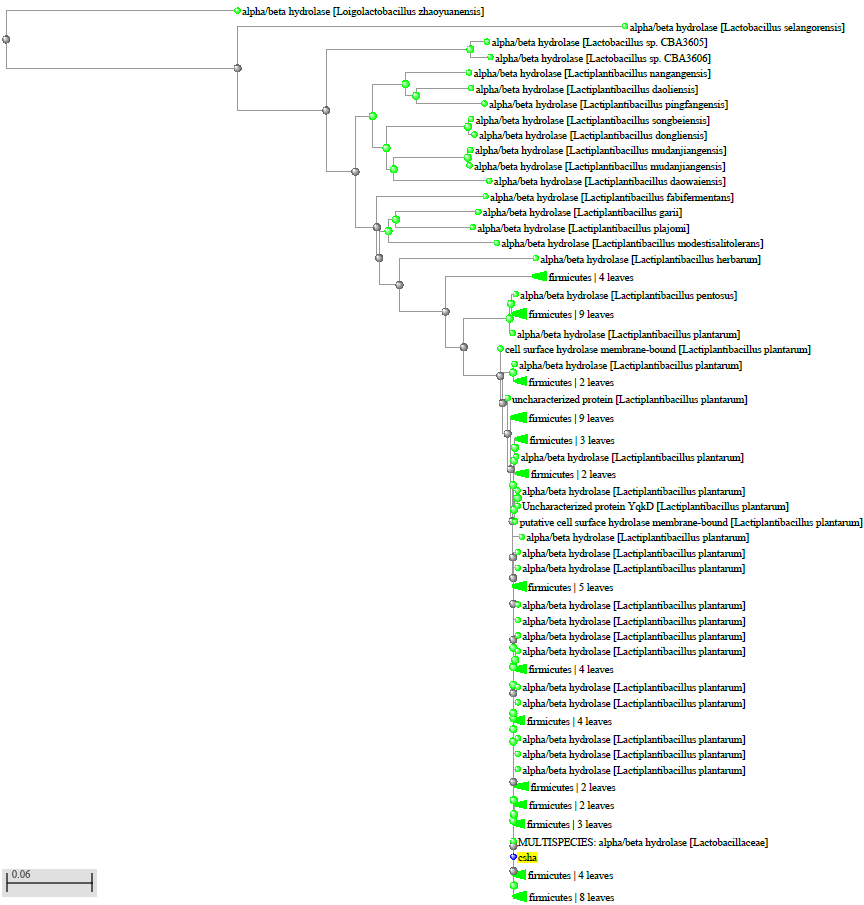
**

**Figure S2**. BLASTp analysis shows that CshA is present in *Lactiplantibacillus, Lactobacillus* and *Loigolactobacillus* genera.


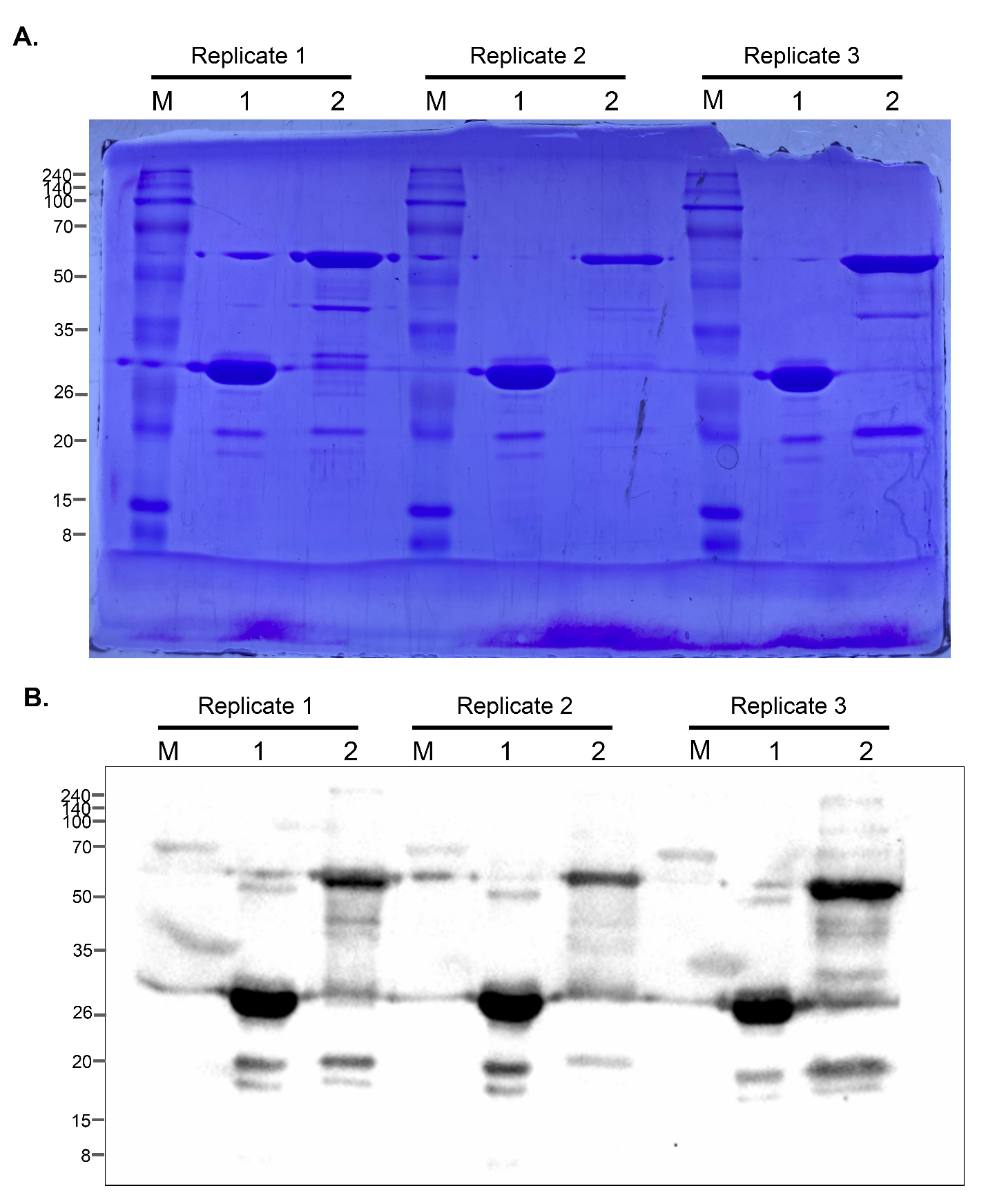


**Figure S3**. Full-length images for the SDS-PAGE and western blot, including replicates. M, marker; 1, sfGFP; 2, CshA-sfGFP. No enhancements were done to the images.
